# Increasing disparities in obesity and severe obesity prevalence among public elementary and middle school students in New York City, school years 2011–12 through 2019–20

**DOI:** 10.1371/journal.pone.0302099

**Published:** 2024-05-15

**Authors:** Kira L. Argenio, Sophia E. Day, Emily M. D’Agostino, Cody Neshteruk, Brooke E. Wagner, Kevin J. Konty

**Affiliations:** 1 New York City Department of Health and Mental Hygiene, Office of School Health, New York, New York, United States of America; 2 Department of Population Health Sciences, Duke University School of Medicine, Durham, North Carolina, United States of America; 3 Duke Center for Childhood Obesity Research, Duke University School of Medicine, Durham, North Carolina, United States of America; 4 Department of Orthopaedic Surgery, Duke University School of Medicine, Durham, North Carolina, United States of America; 5 Duke Clinical Research Institute, Duke University School of Medicine, Durham, North Carolina, United States of America; 6 Duke Global Health Institute, Duke University School of Medicine, Durham, North Carolina, United States of America; New York University Grossman School of Medicine, UNITED STATES

## Abstract

Recent national trends in the United States indicate a significant increase in childhood obesity, a major public health concern with documented physical and mental comorbidities and sociodemographic disparities. We aimed to estimate the prevalence of obesity and severe obesity among youth in New York City (NYC) before the COVID-19 pandemic and examine time trends overall and by key characteristics. We included all valid height and weight measurements of kindergarten through 8^th^ grade public school students aged 5 to 15 from school years 2011–12 through 2019–20 (N = 1,370,890 unique students; 5,254,058 observations). Obesity and severe obesity were determined using age- and sex-specific body mass index percentiles based on the Centers for Disease Control and Prevention growth charts. Analyses were performed using multivariate logistic regression models with repeated cross-sectional observations weighted to represent the student population for each year and clustered by student and school. Among youth attending public elementary and middle schools in NYC, we estimate that 20.9% and 6.4% had obesity and severe obesity, respectively, in 2019–20. While consistent declines in prevalence were observed overall from 2011–12 to 2019–20 (2.8% relative decrease in obesity and 0.2% in severe obesity, *p*<0.001), increasing trends were observed among Black, Hispanic, and foreign-born students, suggesting widening disparities. Extending previous work reporting prevalence estimates in this population, nearly all groups experienced significant increases in obesity and severe obesity from 2016–17 to 2019–20 (relative change = 3.5% and 6.7%, respectively, overall; *p*<0.001). Yet, some of the largest increases in obesity were observed among those already bearing the greatest burden, such as Black and Hispanic students and youth living in poverty. These findings highlight the need for greater implementation of equity-centered obesity prevention efforts. Future research should consider the influence of the COVID-19 pandemic and changes in clinical guidance on childhood obesity and severe obesity in NYC.

## Introduction

Childhood obesity is a major public health concern associated with chronic health conditions and adverse mental health outcomes into adulthood [1–4]. Identifying trends in obesity prevalence is critical to recognize vulnerable youth subgroups and target areas for intervention. Recent national estimates reveal a significant increase in obesity prevalence in the United States (US) from 1999–2000 to 2017–2018 [5]. Specifically, Ogden et al. (2020) reported a significant prevalence increase in children aged 6–11 from 15.8% to 19.3%, and adolescents aged 12–19 from 16.0% to 20.9%, with severe obesity rising from 5.3% to 7.6%. Notably, significant increases in obesity and severe obesity occurred among non-Hispanic Black and Mexican American adolescents [5]. In a similar US study, youth aged 8–11 experienced no significant changes, yet the mean body mass index (BMI; kg/m^2^) from 1999–2006 to 2011–2018 increased significantly among US males and females aged 12–15 and females aged 16–19 [6]. Research has also demonstrated disparities in childhood obesity prevalence related to socioeconomic factors, with the highest risk of obesity among the most socioeconomically disadvantaged children [7, 8].

In contrast to national data, New York City (NYC) public schools, the largest US school system, experienced a significant decline from 21.5% to 20.2% in obesity and 6.4% to 6.0% in severe obesity prevalence among elementary and middle school youth from 2011–12 to 2016–17 school years. These shifts followed a 5.5% and 9.5% relative decline in obesity and severe obesity, respectively, from 2006–07 to 2010–11 [9–12]. However, decreasing trends revealed increasing disparities among subgroups, with smaller declines among Hispanic and Black students, foreign-born youth, and those living in very-poor neighborhoods [10].

This study extends previous works by presenting recent estimates of obesity and severe obesity prevalence in New York City (NYC) public school students from 2011–12 to 2019–20, with a specific focus on analyzing trends from 2016–17 to 2019–20. We further examine trends by student- and school-level characteristics, considering significant disparities previously observed [10]. NYC presents a unique geographic and sociodemographic context and offers rich data from a well-characterized body mass index surveillance system which has been used previously to examine childhood obesity prevalence and trends in the largest US school district. NYC was initially among the cities most impacted by COVID-19 and research suggests significant changes in obesity since the beginning of the pandemic [10, 13–15]. Additionally, the majority of NYC public school students identify as Black or Hispanic and more than 70% of students are economically disadvantaged [9]. Thus, it is crucial to better characterize obesity prevalence and disparities among children in NYC prior to the COVID-19 period to fully evaluate trends and inequity. This work will inform the development of obesity-related policies and programs in NYC as well as other large urban settings.

## Materials and methods

This study utilized data maintained by the NYC Office of School Health (OSH), a joint program of the NYC Department of Education (DOE) and Department of Health and Mental Hygiene (DOHMH). OSH oversees a surveillance system which is used to monitor childhood obesity utilizing data collected through the NYC Fitnessgram program. These data contain annual child-level height and weight measurements for public elementary and middle school students aged 5–15 [16]. The study population included general education students from 2011–12 to 2019–20, excluding those in special education or charter schools. Data and procedures are described in greater detail elsewhere [10–13, 17–19]. The NYC DOHMH Institutional Review Board determined this study to be public health surveillance and therefore “deemed to not be research” (45 CFR 46.102.l.2) and exempt from the requirement for obtaining written informed consent.

Student-level demographic characteristics were obtained from NYC DOE enrollment data and linked to Fitnessgram records using a unique identifier. Demographics included parent/guardian-reported date of birth, gender identity, race/ethnicity, place of birth, and language spoken at home. Four race/ethnicity categories were used: Hispanic, non-Hispanic Black (“Black”), non-Hispanic White (“White”), and Asian/Pacific Islander (“Asian”). Students not reporting Hispanic, non-Hispanic Black, non-Hispanic White, or Asian/Pacific Islander race/ethnicity in a school year are classified as “other,” which includes those reporting multiple races, parent refusal, or missing data. While a distinct racial classification, American Indian/Native Alaskan students are also grouped as “other” due to the small sample size. Although the classification of the “other” race/ethnicity category is not coherent, we use it for modeling and analysis with the aim to reduce its use [13]. In consideration of these limitations, prevalence estimates for students classified as “other” race/ethnicity are not provided. Home/native language was categorized as English, Spanish, or another language, and students’ place of birth was classified as US-born or foreign-born. Students’ socioeconomic status (SES) was determined using geocoded home addresses and the American Community Survey five-year 2012–2018 data, following DOHMH guidelines [20]. Specifically, SES was defined according to the percentage of households living below the federal poverty level (FPL) within a census tract. Students’ home neighborhoods were classified as very-wealthy (<10%), wealthy (10% to <20%), poor (20% to <30%), or very-poor (≥30%) [21].

Primary outcomes were obesity and severe obesity, classified using age- and sex-specific BMI percentiles based on the Centers for Disease Control and Prevention (CDC) growth charts current at data collection [22]. Obesity was defined as a BMI at or above the 95th percentile, while severe obesity was defined as a BMI at or above 120% of the 95th percentile. Obesity includes students with severe obesity. Gender was used in place of sex, according to previous analyses [10–12]. Age in months was calculated, and biologically implausible values (BIVs) were excluded according to the CDC’s criteria and previous NYC analyses [10–13, 23, 24]. From 2011–12 to 2019–20, 5,254,058 valid BMI measurements were analyzed, representing data from 1,370,890 unique students. Specifically, 2,142,860 valid BMI measurements from 866,341 unique students were included from 2016–17 to 2019–20. If all available cases had valid measurements, the data would consist of 5,811,632 observations for K-8 public school students across all school years, with 2,522,386 observations from 2016–17 to 2019–20. Missing data accounted for 9.3% of observations (n = 539,388) from 2011–12 to 2019–20 and 14.7% from 2016–17 to 2019–20 (n = 371,872), the majority (72%) attributable to missingness in the 2019–20 school year due to public schools closing on March 16, 2020 in response to the COVID-19 pandemic. Additionally, 0.3% of observations in each school year were considered BIV (n = 18,186 overall; n = 7,654 from 2016–17 to 2019–20). While some students had valid measurements recorded for all school years, the number of measurements per student ranged from one to nine annual measurements.

Methodology for all current analyses was consistent with Day et al. (2020) [10–12]. We weighted valid student BMI observations (n = 5,254,058) to represent all cases in the K-8 public school student enrollment population within each school year from 2011–12 through 2019–20, using a raking process described in the original study [10]. Estimates of obesity and severe obesity prevalence were calculated using SAS with the SURVEYFREQ procedure. The Wald χ2 statistic determined significant differences in prevalence across student demographic subgroups. To assess whether trends in obesity and severe obesity prevalence were significant across school years 2011–12 to 2019–20 and 2016–17 to 2019–20, four binary logit models were built using SAS PROC SURVEYLOGISTIC, including a linear term for time (index from 0–8 or 0–3). Additionally, several multivariate logistic models were run with repeated cross-sectional observations clustered on the student-level and students clustered by school to examine time trends in severe obesity and obesity prevalence by gender, race/ethnicity, place of birth, home language, and neighborhood SES. We then added an interaction term between time and the corresponding demographic variable to the respective model to assess relative differences in trends across subgroups of each demographic characteristic. All models adjusted for age in months, gender, race/ethnicity, place of birth, home language (English vs. non-English), and school borough by Neighborhood Health Action Center-status and included an interaction term for age in months*gender*race/ethnicity to account for expected growth trajectories. All analyses used SAS version 9.4 (SAS Institute Inc., Cary, North Carolina) with a significance level of α = 0.05 and two-sided *p*-values.

## Results

Table 1 presents valid cases and demographics for each school year. The sample comprised mainly K-5 students (67.5%-69.2%), with slightly more male (50.9%-51.2%) and Hispanic students (40.5%-41.8%). Most students were US-born (84.6%-87.6%) and spoke English (57.0%-58.5%) or Spanish (21.9%-24.0%) at home. About half lived in poor (22.9%-24.4%) or very-poor (23.5%-27.9%) neighborhoods.

**Table 1:** Demographic characteristics overall and by school year for the enrollment population of public school students with valid Fitnessgram body mass index measurements in grades K-8, New York City, 2011–12 to 2019–20.

Characteristic	School Year, %								
	2011–12	2012–13	2013–14	2014–15	2015–16	2016–17	2017–18	2018–19	2019–20
**Total observations, % (n)**	100.0 (626,821)	100.0 (619,611)	100.0 (627,506)	100.0 (618,754)	100.0 (618,506)	100.0 (608,113)	100.0 (603,485)	100.0 (593,612)	100.0 (337,650)
**Gender**									
Female	48.96	49.03	49.03	49.03	48.95	48.91	48.89	48.8	49.07
Male	51.04	50.97	50.97	50.97	51.05	51.09	51.11	51.2	50.93
**Race/ethnicity** ^**a**^									
Asian/Pacific Islander	16.16	16.62	16.93	17.33	17.72	18.23	18.63	18.85	21.5
Non-Hispanic Black	25.98	24.95	23.88	22.78	21.99	21.18	20.6	20.01	17.21
Hispanic	41.05	41.2	41.47	41.78	41.62	41.49	41.55	41.47	40.51
Non-Hispanic White	15.77	15.98	16.26	16.47	16.79	17.02	16.92	17.16	18.09
**Grade type**									
K-5	68.25	68.43	68.7	68.8	69.17	68.77	68.25	67.48	68.77
6–8	31.75	31.57	31.3	31.2	30.83	31.23	31.75	32.52	31.23
**Home-neighborhood SES** ^**b**^									
Very-wealthy (<10%)	20.48	20.67	21.01	21.32	21.63	22.05	22.29	22.84	24.09
Wealthy (10% to <20%)	27.17	27.15	27.16	27.3	27.56	27.75	28.05	28.13	29.53
Poor (20% to <30%)	24.39	24.22	24.15	23.98	23.93	23.74	23.54	23.32	22.87
Very-poor (≥30%)	27.93	27.93	27.65	27.36	26.85	26.42	26.07	25.67	23.49
**Place-of-birth**									
US-born	87.26	87.56	87.36	87.16	86.85	85.99	85.48	85.11	84.64
Foreign-born	12.7	12.39	12.59	12.78	13.08	13.95	14.46	14.82	15.29
**Language spoken at home**									
English	58.37	58.28	58.23	57.93	58.08	57.98	58.01	58.47	56.98
Spanish	24.02	23.82	23.63	23.65	23.19	22.9	22.66	22.26	21.91
Other	17.61	17.9	18.14	18.42	18.73	19.12	19.33	19.27	21.11

Abbreviation: SES, socioeconomic status.

^a^ Students not reporting Hispanic, non-Hispanic Black, non-Hispanic White, or Asian/Pacific Islander race/ethnicity in a school year are classified as “other,” which includes those reporting multiple races, parent refusal, or missing data. While a distinct racial classification, American Indian/Native Alaskan students are also grouped as “other” due to the small sample size. Although the classification of the “other” race/ethnicity category is not coherent, we use it for modeling and analysis with the aim to reduce its use. In consideration of these limitations, prevalence estimates for students classified as “other” race/ethnicity are not provided.

^b^ Percentage of households in the students’ home census tract living below the federal poverty level (FPL) as defined by the 2010 US Census: very-wealthy (<10% of households living below FPL), wealthy (10 to <20% below FPL), poor (20% to <30% below FPL), and very-poor (≥30% below FPL).

### Obesity prevalence

From 2011–12 to 2019–20, the overall prevalence of obesity significantly declined 2.8% (21.5% to 20.9%, *p*<0.001; Table 2), with a significant decrease observed among females (*p*<0.001) but not males. A significant decrease was experienced by students living in wealthy or very-wealthy neighborhoods (*p*<0.001) but not in poor or very-poor neighborhoods. Obesity prevalence decreased among Asian, White, and US-born students (*p*<0.001) but increased among Black, Hispanic, and foreign-born students (*p*<0.05). Obesity increased among students who spoke Spanish at home (*p*<0.001) but decreased among students who spoke English or another language (*p*<0.001). Prevalence also significantly declined among elementary school students (*p*<0.001) but increased among middle school students (*p*<0.05).

**Table 2:** Prevalence^a^ of obesity^b^ among public school youth in grades K-8 by school year and selected characteristics, New York City, 2011–12 to 2019–20.

Characteristic	School Year									Relative change from 2011–12 (%)	Adjusted test for trend (*p*-value)	Relative change from 2016–17 (%)	Adjusted test for trend (*p*-value)
	2011–12	2012–13	2013–14	2014–15	2015–16	2016–17	2017–18	2018–19	2019–20				
	Estimate (95% CI)												
**Total**	21.5 (21.1, 21.9)	21.4 (21.0, 21.8)	21.8 (21.3, 22.2)	20.5 (20.1, 20.9)	20.4 (20.0, 20.8)	20.2 (19.8, 20.7)	20.6 (20.2, 21.1)	20.9 (20.4, 21.3)	20.9 (20.3, 21.5)	-2.8	**<0.001**	3.5	**<0.001**
**Gender**										**	** **	*	** **
Female	19.2 (18.8, 19.6)	19.1 (18.7, 19.6)	19.5 (19.0, 19.9)	18.2 (17.8, 18.7)	18.1 (17.7, 18.5)	17.8 (17.3, 18.2)	18.1 (17.6, 18.6)	18.2 (17.7, 18.7)	18.2 (17.6, 18.8)	-5.2	**<0.001**	2.2	**<0.001**
Male	23.7 (23.3, 24.2)	23.6 (23.1, 24.0)	24.0 (23.5, 24.5)	22.7 (22.2, 23.1)	22.6 (22.1, 23.0)	22.5 (22.1, 23.0)	23.0 (22.5, 23.5)	23.4 (22.9, 23.9)	23.5 (22.9, 24.2)	-0.8	0.083	4.4	**<0.001**
**Race/ethnicity**										***	** **	**	** **
Asian/Pacific Islander	13.7 (13.2, 14.3)	13.8 (13.2, 14.3)	14.4 (13.8, 15.0)	13.6 (13.1, 14.2)	13.6 (13.0, 14.1)	13.1 (12.6, 13.7)	13.2 (12.7, 13.8)	13.5 (12.9, 14.0)	13.3 (12.6, 14.0)	-2.9	**<0.001**	1.5	0.082
Non-Hispanic Black	22.4 (22.0, 22.7)	22.0 (21.7, 22.4)	22.4 (22.0, 22.7)	21.5 (21.2, 21.8)	21.3 (21.0, 21.7)	21.6 (21.2, 21.9)	22.1 (21.7, 22.5)	22.6 (22.2, 23.0)	23.0 (22.4, 23.5)	2.7	**0.022**	6.5	**<0.001**
Hispanic	26.5 (26.0, 26.9)	26.6 (26.2, 27.1)	27.3 (26.8, 27.7)	25.7 (25.3, 26.0)	25.7 (25.3, 26.1)	25.8 (25.4, 26.2)	26.4 (26.0, 26.8)	26.8 (26.4, 27.1)	27.0 (26.5, 27.5)	1.9	**0.004**	4.7	**<0.001**
Non-Hispanic White	15.4 (14.6, 16.1)	15.0 (14.2, 15.8)	15.0 (14.2, 15.7)	13.7 (12.9, 14.4)	13.6 (12.8, 14.3)	13.0 (12.3, 13.7)	13.4 (12.6, 14.1)	13.3 (12.6, 14.1)	13.3 (12.5, 14.2)	-13.6	**<0.001**	2.3	**0.004**
**Grade type**										***	** **	***	** **
K-5	21.3 (20.8, 21.8)	21.2 (20.7, 21.7)	21.8 (21.3, 22.3)	20.3 (19.8, 20.8)	20.2 (19.6, 20.7)	20.0 (19.4, 20.5)	20.4 (19.9, 21.0)	20.5 (19.9, 21.1)	20.4 (19.7, 21.1)	-4.2	**<0.001**	2.0	**<0.001**
6–8	22.0 (21.3, 22.7)	21.7 (21.0, 22.4)	21.7 (21.0, 22.4)	21.0 (20.3, 21.7)	20.9 (20.2, 21.5)	20.8 (20.1, 21.5)	21.0 (20.3, 21.8)	21.6 (20.8, 22.4)	22.0 (20.9, 23.1)	0.3	**0.042**	5.8	**<0.001**
**Home-neighborhood SES**										***	** **	*	** **
Very-wealthy (<10%)	17.1 (16.3, 17.8)	16.6 (15.8, 17.3)	16.7 (15.9, 17.4)	15.5 (14.8, 16.3)	15.5 (14.8, 16.3)	15.2 (14.5, 16.0)	15.6 (14.9, 16.3)	15.8 (15.0, 16.6)	15.8 (14.8, 16.8)	-7.6	**<0.001**	3.9	**<0.001**
Wealthy (10% to <20%)	20.4 (19.9, 20.9)	20.3 (19.8, 20.8)	20.8 (20.3, 21.4)	19.7 (19.2, 20.2)	19.5 (19.0, 20.0)	19.3 (18.7, 19.8)	19.6 (19.0, 20.1)	19.8 (19.3, 20.4)	19.7 (19.0, 20.4)	-3.4	**<0.001**	2.1	**<0.001**
Poor (20% to <30%)	23.2 (22.7, 23.7)	23.2 (22.7, 23.8)	23.7 (23.2, 24.3)	22.4 (21.8, 22.9)	22.2 (21.6, 22.8)	22.3 (21.7, 22.8)	22.7 (22.1, 23.3)	23.0 (22.4, 23.6)	23.4 (22.7, 24.2)	0.9	0.644	4.9	**<0.001**
Very-poor (> = 30%)	24.4 (24.0, 24.9)	24.3 (23.8, 24.8)	24.9 (24.4, 25.4)	23.5 (23.0, 23.9)	23.5 (23.0, 24.0)	23.5 (23.0, 24.0)	24.1 (23.6, 24.7)	24.6 (24.0, 25.1)	24.9 (24.2, 25.6)	2.0	0.051	6.0	**<0.001**
**Place-of-birth**										***	** **	** **	
US-born	22.2 (21.8, 22.6)	22.0 (21.6, 22.4)	22.4 (21.9, 22.8)	21.1 (20.6, 21.5)	21.0 (20.5, 21.4)	20.8 (20.4, 21.3)	21.2 (20.8, 21.7)	21.5 (21.0, 22.0)	21.5 (20.9, 22.1)	-3.2	**<0.001**	3.4	**<0.001**
Foreign-born	16.9 (16.4, 17.4)	17.1 (16.6, 17.6)	17.7 (17.2, 18.2)	16.7 (16.2, 17.1)	16.5 (16.1, 17.0)	16.6 (16.1, 17.1)	16.9 (16.4, 17.4)	17.4 (16.9, 17.9)	17.6 (17.0, 18.2)	4.1	**0.025**	6.0	**<0.001**
**Language spoken at home**										***	** **	*	** **
English	21.3 (20.9, 21.7)	21.0 (20.6, 21.5)	21.3 (20.8, 21.7)	20.2 (19.7, 20.6)	20.0 (19.6, 20.5)	19.9 (19.4, 20.3)	20.3 (19.8, 20.7)	20.5 (20.0, 21.0)	20.6 (19.9, 21.2)	-3.3	**<0.001**	3.5	**<0.001**
Spanish	27.3 (26.8, 27.9)	27.7 (27.2, 28.2)	28.5 (27.9, 29.0)	26.6 (26.2, 17.1)	26.7 (26.2, 27.2)	26.9 (26.4, 27.3)	27.6 (27.1, 28.1)	27.9 (27.5, 28.4)	28.2 (27.6, 28.8)	3.3	**<0.001**	4.8	**<0.001**
Other	14.2 (13.7, 14.6)	14.1 (13.7, 14.6)	14.7 (14.2, 15.2)	13.7 (13.2, 14.2)	13.7 (13.2, 14.1)	13.2 (12.8, 13.7)	13.4 (13.0, 13.9)	13.6 (13.2, 14.1)	13.5 (12.9, 14.1)	-4.9	**<0.001**	2.3	**0.028**

Abbreviation: SES, socioeconomic status.

^a^ Prevalence estimates were based on valid body mass index (BMI) (kg/m^2^) measurements weighted to be representative of the entire population of public school students in grades K-8 in a given school year by race/ethnicity, school borough by Neighborhood Health Action Center (NHAC) neighborhood (neighborhoods defined by low-income and disproportionate rates of morbidity and mortality), free/reduced price meal status, grade, gender, age, and school type (elementary vs. middle). Prevalence estimates of obesity reflect the enrollment population.

^b^ Obesity is defined as having a BMI for age and sex at or above the 95th percentile according to the CDC’s 2000 growth charts. Students having at least one measure for height, weight, weight-for-height, or BMI that was identified as biologically implausible by the 2000 CDC growth chart Z-score and the World Health Organization’s fixed exclusion criteria were excluded from the measured population.

^c^ To test for trends over school years, multivariate models were built that included a linear term for trend, along with gender, age, race/ethnicity, school borough by NHAC neighborhoods, free/reduced price meal status, place-of-birth, language spoken at home, and an interaction by age, gender, and race/ethnicity as covariates. Both school and student identifiers were used as cluster variables.

^d^ Students not reporting Hispanic, non-Hispanic Black, non-Hispanic White, or Asian/Pacific Islander race/ethnicity in a school year are classified as “other,” which includes those reporting multiple races, parent refusal, or missing data. While a distinct racial classification, American Indian/Native Alaskan students are also grouped as “other” due to the small sample size. Although the classification of the “other” race/ethnicity category is not coherent, we use it for modeling and analysis with the aim to reduce its use. In consideration of these limitations, prevalence estimates for students classified as “other” race/ethnicity are not provided.

^e^ Percentage of households in the students’ home census tract living below the federal poverty level (FPL) as defined by the 2010 US Census: very-wealthy (<10% of households living below FPL), wealthy (10 to <20% below FPL), poor (20% to <30% below FPL), and very-poor (≥30% below FPL).

^***^Relative differences across subgroups *p*<0.001

^**^p<0.01

^*^p<0.05.

**Fig 1:**
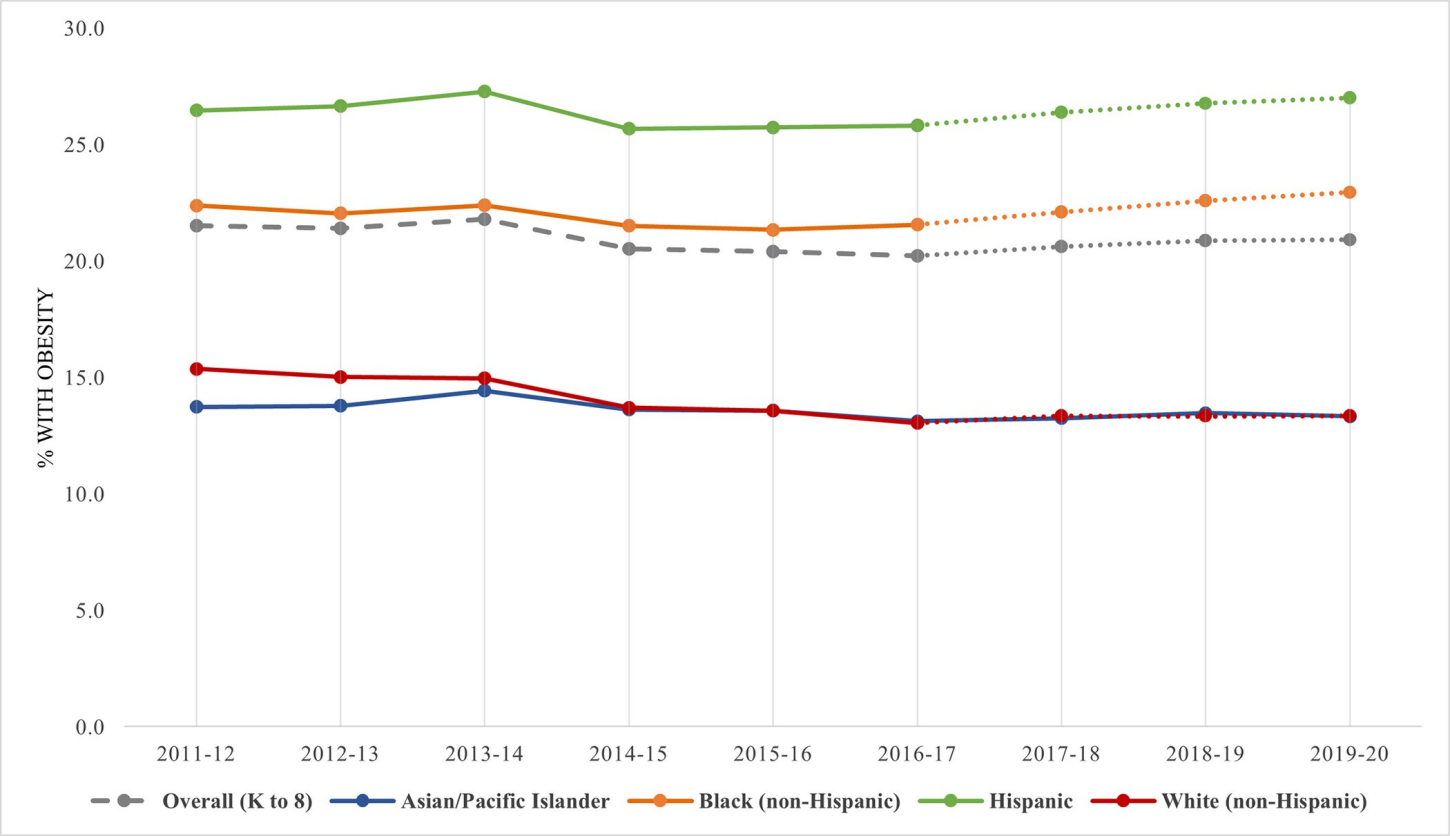
Obesity trends over time by race/ethnicity. Adjusted binary logit models were built, including a linear term for time (index from 0–8 or 0–3), to assess whether trends in obesity and severe obesity prevalence were significant across school years 2011–12 to 2019–20 and 2016–17 to 2019–20. Models were run for all NYC public elementary and middle school students, which were significant at *p*<0.001 for both time periods, and were also run separately for each race/ethnicity subgroup (*p*<0.05 for all subgroups from 2011–12 to 2019–20 and *p*<0.01 for all except Asian/Pacific Islander students from 2016–17 to 2019–20, n.s.). Models were weighted and clustered by school and student.

From 2016–17 to 2019–20, the overall prevalence of obesity significantly increased 3.5% (20.2% to 20.9%, *p*<0.001; Table 2), with significant increases observed across gender, grade type, place of birth, home language, and home-neighborhood SES subgroups (*p*<0.05). Significant increases were observed among Black, Hispanic, and White students (*p*<0.01) but not among Asian students (difference in trends among subgroups *p*<0.01). Although White students experienced a significant relative increase, the change was less than half that observed among Black students (2.3% vs. 6.5%, both *p*<0.01; Fig 1). Specifically, the absolute difference in prevalence between Black and White students increased from 8.6% to 9.7% (1.13 times increase). Hispanic students experienced a smaller increase in obesity (25.8% to 27.0%, *p*<0.001) than Black students (21.6% to 23.0%, *p*<0.001), but obesity prevalence remained highest among Hispanic students. Obesity prevalence increased significantly among both K-5 and 6–8 grade youth, with a larger increase observed among middle school students (5.8% increase to 22.0%, *p*<0.001) than elementary school students (2.0% increase to 20.4%, *p*<0.001). Females experienced only half the relative increase in obesity prevalence of males (2.2% vs. 4.4%, both *p*<0.001; difference in trends *p* = 0.04).

From 2016–17 to 2019–20, a larger relative increase in obesity (4.8% vs. 3.5%) was experienced by Spanish-speaking than among English-speaking youth (26.9% to 28.2% vs. 19.9% to 20.6%, respectively, both *p*<0.001). Despite an increasing trend, obesity prevalence consistently remained lowest, and the smallest increase (2.3%) was observed, among students who spoke a language other than English or Spanish at home (13.2% to 13.5%, *p* = 0.03). Whereas obesity prevalence increased significantly (3.4% relative increase) among students born in the US (20.8% to 21.5% from 2016–17 to 2019–20, *p*<0.001), foreign-born youth experienced a larger relative change (16.6% to 17.6%, 6.0% relative increase, from 2016–17 to 2019–20, *p*<0.001). However, the relative difference in changes between place-of-birth subgroups was not significant (*p* = 0.64). Additionally, obesity remained higher among US-born students.

Larger increases in obesity prevalence from 2016–17 to 2019–20 were experienced by students living in poor (4.9% relative increase; 22.3% to 23.4%, *p*<0.001) or very-poor neighborhoods (23.5% to 24.9%, 6.0% increase, *p*<0.001) as compared to students living in very-wealthy neighborhoods (15.2% to 15.8%, 3.9% increase, *p*<0.001), while the smallest increases were observed among students living in wealthy neighborhoods (19.3% to 19.7%, 2.1% increase, *p*<0.001). The disparity in obesity prevalence between students living in very-poor neighborhoods as compared to those in very-wealthy neighborhoods increased from 8.3% to 9.1% (1.10 times increase). Across home language and neighborhood poverty subgroups, relative differences in trends were significant (*p* = 0.04 and *p* = 0.02, respectively).

### Severe obesity prevalence

From 2011–12 to 2019–20, the overall prevalence of severe obesity significantly declined 0.2% (6.398% to 6.387%, *p*<0.001; Table 3). Males experienced a significant increase in severe obesity (*p*<0.001), while no significant changes were observed among females. Severe obesity increased significantly among youth in poor or very-poor neighborhoods (*p*<0.001) but not those in wealthy or very-wealthy neighborhoods. Prevalence significantly declined among White students, but an increasing trend was observed among Black and Hispanic students (*p*<0.001). Similarly, severe obesity significantly decreased among English-speakers (*p*<0.05) but increased among Spanish-speaking students (*p*<0.001). US-born students experienced a significant decline in severe obesity (*p*<0.01), while prevalence increased significantly among foreign-born students (*p*<0.001). A significant relative increase was also observed among youth in grades 6–8 (*p*<0.001), but the opposite trend was observed among grades K-5 (*p*<0.05).

**Table 3:** Prevalence^a^ of severe obesity^b^ among public school youth in grades K-8 by school year and selected characteristics, New York City, 2011–12 to 2019–20.

Characteristic	School Year									Relative change from 2011–12 (%)	Adjusted test for trend (*p*-value)	Relative change from 2016–17 (%)	Adjusted test for trend (*p*-value)
	2011–12	2012–13	2013–14	2014–15	2015–16	2016–17	2017–18	2018–19	2019–20				
	Estimate (95% CI)												
**Total**	6.4 (6.2, 6.6)	6.3 (6.1, 6.5)	6.4 (6.3, 6.6)	5.9 (5.8, 6.1)	5.9 (5.8, 6.1)	6.0 (5.8, 6.1)	6.2 (6.0, 6.4)	6.4 (6.2, 6.6)	6.4 (6.1, 6.7)	-0.2	**<0.001**	6.7	**<0.001**
**Gender**										***	** **	***	** **
Female	5.4 (5.2, 5.6)	5.4 (5.3, 5.6)	5.5 (5.3, 5.7)	5.1 (4.9, 5.3)	5.0 (4.9, 5.2)	5.0 (4.9, 5.2)	5.1 (4.9, 5.3)	5.2 (5.0, 5.4)	5.2 (5.0, 5.5)	-3.7	0.74	4.0	**<0.001**
Male	7.3 (7.1, 7.5)	7.2 (7.0, 7.4)	7.3 (7.1, 7.5)	6.8 (6.6, 7.0)	6.8 (6.6, 7.0)	6.8 (6.6, 7.0)	7.2 (7.0, 7.4)	7.5 (7.3, 7.7)	7.5 (7.2, 7.8)	2.7	**<0.001**	10.3	**<0.001**
**Race/ethnicity**										***	** **	*	** **
Asian/Pacific Islander	2.4 (2.3, 2.6)	2.5 (2.3, 2.6)	2.6 (2.4, 2.8)	2.4 (2.3, 2.6)	2.4 (2.2, 2.6)	2.3 (2.2, 2.5)	2.5 (2.3, 2.7)	2.6 (2.4, 2.7)	2.5 (2.3, 2.7)	4.2	0.979	8.7	**0.014**
Non-Hispanic Black	7.7 (7.5, 7.9)	7.5 (7.3, 7.8)	7.6 (7.4, 7.8)	7.3 (7.1, 7.5)	7.4 (7.2, 7.6)	7.5 (7.3, 7.7)	7.8 (7.6, 8.0)	8.2 (8.0, 8.5)	8.2 (7.8, 8.5)	6.5	**<0.001**	9.3	**<0.001**
Hispanic	8.1 (7.9, 8.3)	8.1 (7.9, 8.3)	8.4 (8.2, 8.6)	7.7 (7.6, 7.9)	7.8 (7.6, 8.0)	7.9 (7.7, 8.1)	8.3 (8.1, 8.5)	8.6 (8.4, 8.8)	8.7 (8.5, 9.0)	7.4	**<0.001**	10.1	**<0.001**
Non-Hispanic White	3.8 (3.5, 4.1)	3.9 (3.6, 4.1)	3.7 (3.4, 4.0)	3.4 (3.1, 3.6)	3.4 (3.1, 3.7)	3.3 (3.0, 3.5)	3.3 (3.0, 3.5)	3.3 (3.1, 3.6)	3.3 (3.0, 3.7)	-13.2	**<0.001**	2.0	0.123
**Grade type**										** **	** **	** **	** **
K-5	6.1 (5.9, 6.3)	6.0 (5.8, 6.2)	6.2 (6.0, 6.4)	5.7 (5.5, 5.9)	5.7 (5.5, 5.9)	5.7 (5.5, 5.9)	6.0 (5.7, 6.2)	6.1 (5.9, 6.3)	6.0 (5.7, 6.3)	-1.6	**0.017**	5.3	**<0.001**
6–8	7.1 (6.7, 7.5)	7.0 (6.6, 7.3)	6.9 (6.6, 7.3)	6.6 (6.3, 6.9)	6.5 (6.2, 6.8)	6.5 (6.2, 6.9)	6.7 (6.3, 7.0)	7.0 (6.6, 7.4)	7.1 (6.6, 7.7)	0.5	**<0.001**	9.2	**<0.001**
**Home-neighborhood SES**										***	** **	**	** **
Very-wealthy (<10%)	4.5 (4.2, 4.8)	4.4 (4.1, 4.6)	4.4 (4.2, 4.7)	4.1 (3.8, 4.4)	4.1 (3.8, 4.4)	4.0 (3.8, 4.3)	4.1 (3.9, 4.4)	4.2 (3.9, 4.5)	4.2 (3.8, 4.5)	-6.7	0.253	5.0	**0.009**
Wealthy (10% to <20%)	5.7 (5.5, 5.9)	5.6 (5.4, 5.8)	5.7 (5.5, 6.0)	5.4 (5.2, 5.6)	5.3 (5.1, 5.5)	5.3 (5.1, 5.5)	5.5 (5.2, 5.7)	5.7 (5.4, 5.9)	5.6 (5.3, 5.9)	-1.8	0.096	5.7	**<0.001**
Poor (20% to <30%)	6.8 (6.6, 7.1)	6.9 (6.7, 7.1)	7.0 (6.8, 7.3)	6.5 (6.2, 6.7)	6.6 (6.3, 6.8)	6.7 (6.4, 7.0)	6.9 (6.6, 7.2)	7.2 (6.9, 7.5)	7.4 (7.1, 7.8)	8.8	**<0.001**	10.4	**<0.001**
Very-poor (> = 30%)	8.1 (7.8, 8.4)	8.0 (7.7, 8.2)	8.1 (7.9, 8.4)	7.5 (7.2, 7.7)	7.6 (7.3, 7.8)	7.6 (7.3, 7.8)	8.0 (7.7, 8.3)	8.4 (8.1, 8.6)	8.4 (8.0, 8.8)	3.7	**<0.001**	10.5	**<0.001**
**Place-of-birth**										***	** **	** **	
US-born	6.8 (6.6, 7.0)	6.7 (6.5, 6.9)	6.8 (6.6, 7.0)	6.3 (6.1, 6.4)	6.3 (6.1, 6.5)	6.3 (6.1, 6.5)	6.5 (6.3, 6.7)	6.8 (6.5, 7.0)	6.7 (6.4, 7.0)	-1.5	**0.009**	6.3	**<0.001**
Foreign-born	3.8 (3.6, 4.0)	3.9 (3.7, 4.1)	4.0 (3.8, 4.2)	3.8 (3.6, 4.0)	3.8 (3.6, 3.9)	3.9 (3.7, 4.1)	4.0 (3.8, 4.2)	4.3 (4.1, 4.5)	4.5 (4.2, 4.7)	18.4	**<0.001**	15.4	**<0.001**
**Language spoken at home**										***	** **	***	** **
English	6.9 (6.7, 7.1)	6.8 (6.6, 7.0)	6.9 (6.7, 7.1)	6.4 (6.2, 6.6)	6.4 (6.2, 6.6)	6.4 (6.2, 6.6)	6.6 (6.4, 6.8)	6.8 (6.6, 7.0)	6.8 (6.5, 7.1)	-1.4	**0.025**	6.2	**<0.001**
Spanish	7.8 (7.6, 8.1)	7.9 (7.6, 8.1)	8.2 (7.9, 8.4)	7.4 (7.2, 7.6)	7.5 (7.3, 7.7)	7.6 (7.4, 7.9)	8.1 (7.8, 8.3)	8.4 (8.2, 8.6)	8.6 (8.3, 8.9)	10.3	**<0.001**	13.2	**<0.001**
Other	2.8 (2.6, 2.9)	2.7 (2.6, 2.9)	2.8 (2.7, 3.0)	2.6 (2.5, 2.8)	2.6 (2.5, 2.8)	2.6 (2.4, 2.7)	2.6 (2.5, 2.8)	2.7 (2.6, 2.9)	2.6 (2.4, 2.8)	-7.1	0.327	2.0	0.248

Abbreviation: SES, socioeconomic status.

^a^ Prevalence estimates were based on valid body mass index (BMI) (kg/m^2^) measurements weighted to be representative of the entire population of public school students in grades K-8 in a given school year by race/ethnicity, school borough by Neighborhood Health Action Center (NHAC) neighborhood (neighborhoods defined by low-income and disproportionate rates of morbidity and mortality), free/reduced price meal status, grade, gender, age, and school type (elementary vs. middle). Prevalence estimates of obesity reflect the enrollment population.

^b^ Severe obesity is defined as having a BMI at or above 120% of the 95th percentile BMI-for-sex-and-age cut-off according to the CDC’s 2000 growth charts. Students having at least one measure for height, weight, weight-for-height, or BMI that was identified as biologically implausible by the 2000 CDC growth chart Z-score and the World Health Organization’s fixed exclusion criteria were excluded from the measured population.

^c^ To test for trends over school years, multivariate models were built that included a linear term for trend, along with gender, age, race/ethnicity, school borough by NHAC neighborhoods, free/reduced price meal status, place-of-birth, language spoken at home, and an interaction by age, gender, and race/ethnicity as covariates. Both school and student identifiers were used as cluster variables.

^d^ Students not reporting Hispanic, non-Hispanic Black, non-Hispanic White, or Asian/Pacific Islander race/ethnicity in a school year are classified as “other,” which includes those reporting multiple races, parent refusal, or missing data. While a distinct racial classification, American Indian/Native Alaskan students are also grouped as “other” due to the small sample size. Although the classification of the “other” race/ethnicity category is not coherent, we use it for modeling and analysis with the aim to reduce its use. In consideration of these limitations, prevalence estimates for students classified as “other” race/ethnicity are not provided.

^e^ Percentage of households in the students’ home census tract living below the federal poverty level (FPL) as defined by the 2010 US Census: very-wealthy (<10% of households living below FPL), wealthy (10 to <20% below FPL), poor (20% to <30% below FPL), and very-poor (≥30% below FPL).

^***^Relative differences across subgroups *p*<0.001

^**^p<0.01

^*^p<0.05.

From 2016–17 to 2019–20, severe obesity prevalence significantly increased 6.7% (6.0% to 6.4%, *p*<0.001; Table 3), with significant increases observed among gender, grade type, home-neighborhood SES, and place-of-birth subgroups (*p*<0.01) but not among White students (*p* = 0.12) or students who spoke a language other than English or Spanish at home (*p* = 0.25).

Severe obesity prevalence consistently remained higher in male students, who experienced more than double the relative increase of females (10.3% as compared to 4.0%) from 2016–17 to 2019–20 (6.8% to 7.5% vs. 5.0% to 5.2%, respectively, both *p*<0.001; relative difference in trends, *p*<0.001). Despite a large relative increase (15.4%) among foreign-born youth from 2016–17 to 2019–20 (3.9% to 4.5%), severe obesity remained lower than among US-born youth (6.3% to 6.7%, 6.3% increase; both *p*<0.001). The relative difference in trends between US- vs. foreign-born youth was not significant (*p* = 0.26).

While severe obesity increased significantly across all SES groups, the relative changes in prevalence experienced by students living in poor (10.4% increase) or very-poor (10.5% increase) neighborhoods (6.7% to 7.4% vs. 7.6% to 8.4%, respectively; both *p*<0.001) were more than twice that observed among those in very-wealthy neighborhoods (4.0% to 4.2%, 5.0% increase, *p*<0.01). Severe obesity trends followed a similar pattern among youth living in wealthy neighborhoods (5.3% to 5.6%, 5.7% increase, *p*<0.001). Overall differences in relative changes between SES subgroups were significant (*p*<0.01). Additionally, the absolute difference in prevalence between students in very-poor and very-wealthy neighborhoods increased from 3.6% to 4.2% (1.17 times increase).

**Fig 2:**
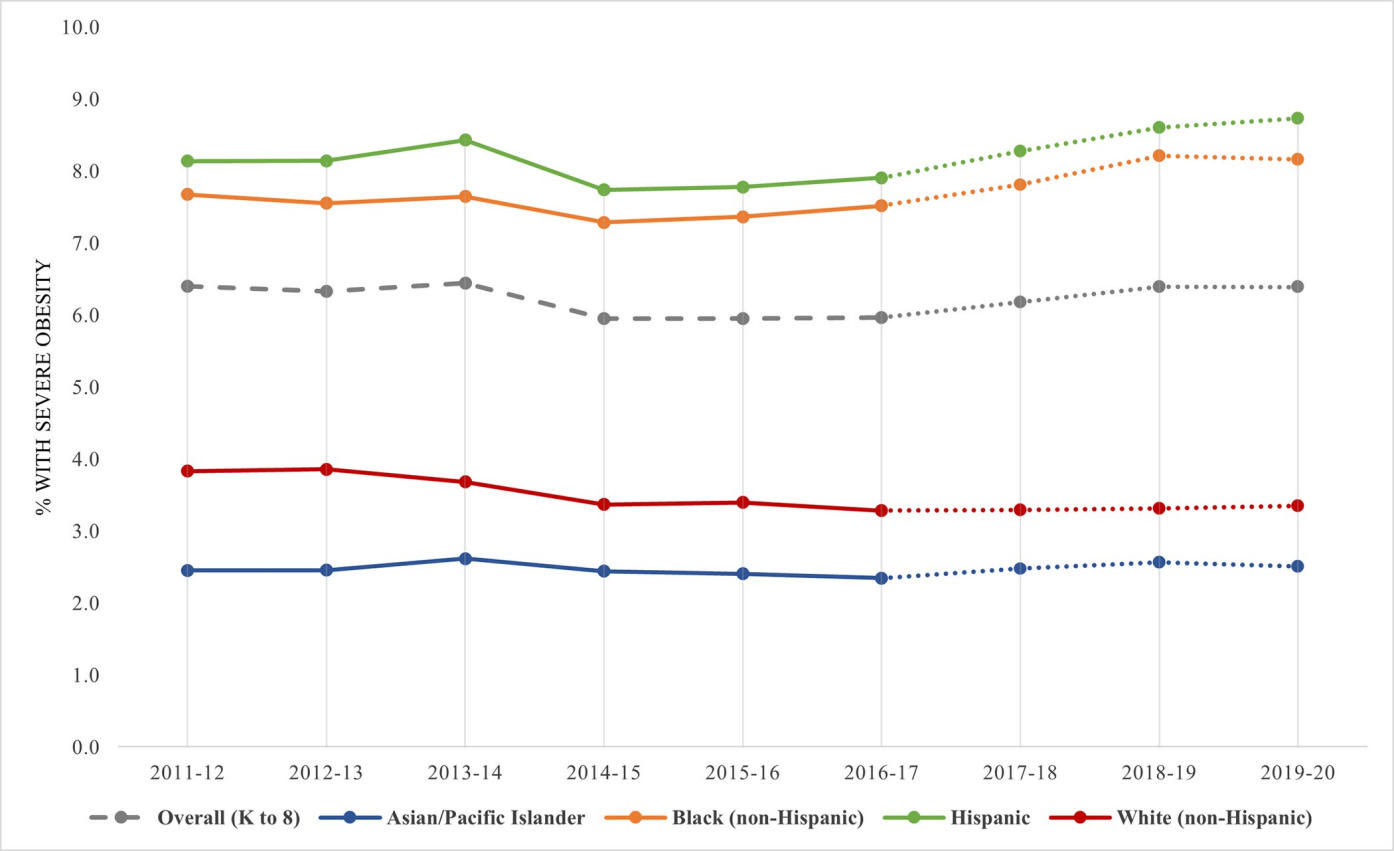
Severe obesity trends over time by race/ethnicity. Adjusted binary logit models were built, including a linear term for time (index from 0–8 or 0–3), to assess whether trends in severe obesity prevalence were significant across school years 2011–12 to 2019–20 and 2016–17 to 2019–20. Models were run for all NYC public elementary and middle school students, which were significant at *p*<0.001 for both time periods, and were also run separately for each race/ethnicity subgroup (*p*<0.001 for all except Asian/Pacific Islander students, n.s., from 2011–12 to 2019–20 and *p*<0.05 for all except non-Hispanic White students from 2016–17 to 2019–20, n.s.). Models were weighted and clustered by school and student.

Across all race/ethnicity subgroups except White students, severe obesity increased significantly from 2016–17 to 2019–20, suggesting an acceleration of already-widening racial/ethnic disparities (Fig 2). Specifically, the difference in relative increases in severe obesity between Asian (8.7%), Black (9.3%), and Hispanic (10.1%) students (2.3% to 2.5%, *p* = 0.01; 7.5% to 8.2%, *p*<0.001; and 7.9% to 8.7%, *p*<0.001, respectively) were more than four-to-five times those observed among White students (2.0% relative increase, *p* = 0.12), with prevalence consistently remaining highest among Hispanic youth. Further, the increase in severe obesity prevalence of Spanish-speaking students more than doubled that of English-speaking students (7.6% to 8.6%, 13.2% increase vs. 6.4% to 6.8%, 6.2% increase, respectively, both *p*<0.001). Still, prevalence remained lowest among students speaking a language other than English or Spanish at home with no significant change in severe obesity prevalence during either time period. Relative changes in severe obesity prevalence differed significantly by race/ethnicity and home language (*p* = 0.04 and *p*<0.001, respectively).

While a larger increase in severe obesity prevalence from 2016–17 to 2019–20 was observed among 6–8 grade students (6.5% to 7.1%, 9.2% increase, *p*<0.001) compared with K-5 grade students (5.7% to 6.0%, 5.3% increase, *p*<0.001), the relative difference in trends between grade types was not significant (*p* = 0.08).

### Obesity prevalence and neighborhood poverty by race/ethnicity, 2019–20

**Fig 3:**
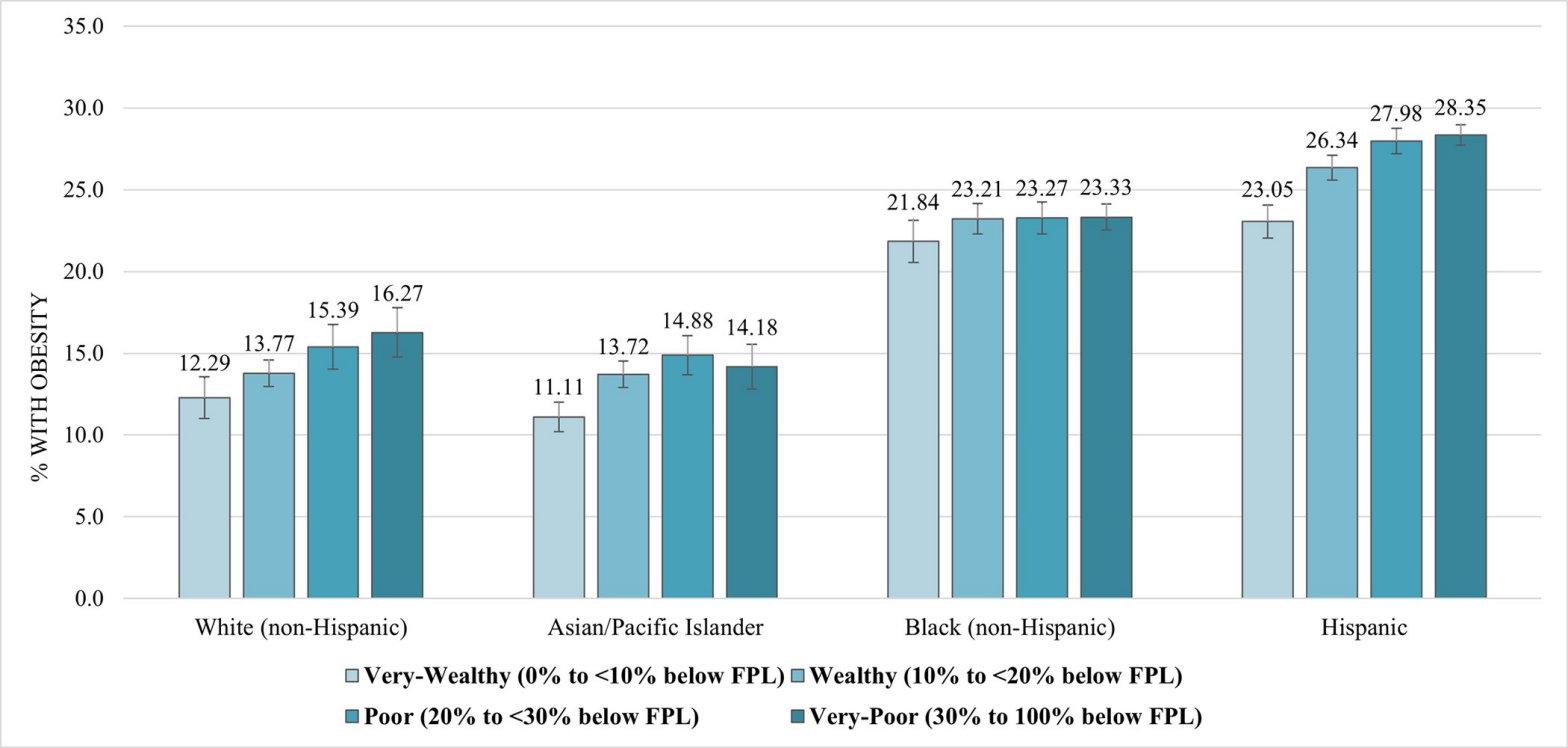
Obesity prevalence of NYC public school K-8 students by race/ethnicity and home-neighborhood poverty level, 2019–20. Race/ethnicity by home-neighborhood poverty interaction was estimated using a logistic model of obesity in the 2019–20 school year and was significant at *p*<0.001. Model was weighted and clustered by school and student.

Obesity prevalence estimates for 2019–20 reflect a significantly increasing trend across home-neighborhood poverty-levels, consistent with previous data for 2016–17. Specifically, obesity increased from very-wealthy to very-poor areas, with students living in very-poor and poor areas experiencing a greater obesity burden. As previously reported, this significant increasing pattern was consistent across all race/ethnicity subgroups (*p*<0.001) except among Black students (*p* = 0.56; Fig 3). Obesity prevalence by poverty-levels differed significantly across race/ethnicity subgroups (*p*<0.001).

## Discussion

In contrast to previously reported declines in obesity and severe obesity prevalence among children in NYC public schools from 2006–07 to 2010–11 [11, 12], these analyses demonstrate significant increasing trends among nearly all subgroups of children from the school year 2016–17 through 2019–20. Despite these changes, overall analyses inclusive of the full study period show obesity and severe obesity prevalence continued to decline from 2011–12 to 2019–20. Still, while the direction of these trends was consistent with data for 2011–12 to 2016–17 alone, relative decreases from 2011–12 to 2019–20 were much smaller (6.0% vs. 2.8% relative decline in obesity and 6.3% vs. 0.2% relative decline in severe obesity, respectively), suggesting a deceleration of continued declines observed among this population [10].

Nationally, some evidence suggests that childhood obesity prevalence increased in the 1980s and 1990s, and plateaued in the early 2000s [25, 26], with no significant changes in youth obesity prevalence reported from 2007–2008 through 2015–2016 [27]. However, the most current nationally representative samples show significant increases in obesity among children aged 6–11 and adolescents aged 12–19, in both obesity and severe obesity from 1999–2000 to 2017–2018 [5]. Additional evidence also suggests the prevalence of obesity increased significantly from 2011–2012 through 2017–2020 among US children aged 2–19 [28]. Thus, more recent national data demonstrating increasing trends appear to follow consistent patterns of obesity prevalence over the past two decades, despite a possible leveling of obesity trends observed through 2015–2016. While contrasting, declining trends have consistently been reported among NYC schoolchildren [10–12], the current analyses of a concentrated period of recent data (2016–17 to 2019–20) showed patterns comparable with national trends.

### Comparison to national trends for subgroups

Consistent with recent national trends, the current data suggest a significant increase in obesity prevalence among non-Hispanic Black students in NYC during both the 2011–12 to 2019–20 and 2016–17 to 2019–20 time periods [5]. Relatedly, Ogden et al. (2020) reported that obesity prevalence increased from 1999–2000 to 2017–2018 among Mexican American children aged 12–19, although not across all Hispanic-origin subgroups. While we could not examine trends by Hispanic ethnicity subgroups, we found that obesity and severe obesity prevalence significantly increased among Hispanic students. Our findings contrast with previously reported NYC trends from 2011–12 through 2016–17, in which we observed significant decreases in obesity prevalence among Black and Hispanic students [10]. While previous declines among Black and Hispanic youth were smaller than among White students, current trends may provide evidence for growing inequity in obesity and severe obesity prevalence.

Additionally, consistent with 2016–17 data [10], we found that obesity prevalence in 2019–20 remained similar across poverty levels among Black students but increased with poverty in all other race/ethnicity subgroups. As previous studies have shown associations between structural and interpersonal racism and obesity, these findings demonstrate the importance of continued public health surveillance [29, 30]. Further, while there was a continued decline in obesity among US-born students in NYC from 2011–12 to 2019–20, foreign-born students experienced a significant increase. Relatedly, larger obesity increases were observed among foreign- than US-born youth from 2016–17 to 2019–20. Despite prevalence remaining highest among US-born students, findings suggest narrowing of the gap between place-of-birth subgroups, which global changes in childhood obesity or possible acculturation effects may explain [31, 32].

In contrast to an analysis of national data from 2011–2012 to 2017–2020, which showed significant increases in obesity prevalence among boys and girls aged 2–19, we found that obesity prevalence decreased only among females in NYC from 2011–12 to 2019–20 [28]. Relatedly, while Hu et al. (2022) reported significant overall obesity increases among US children aged 2–19 from 2011–2012 to 2017–2020, a significant overall decrease was experienced by NYC students aged 5–15 from 2011–12 through 2019–20 [28]. Although NYC results stratified by grade type showed a significant decrease in obesity among elementary school students, national data show no significant change in obesity among youth aged 6–11 [28]. In contrast to previous NYC-specific and recent national findings, we observed a significant increase in severe obesity prevalence among male (and not female) students from 2011–12 through 2019–20, although severe obesity increased significantly among both male and female students from 2016–17 to 2019–20. While there are likely true national vs. regional differences in childhood obesity by gender, design considerations such as periods analyzed, single- vs. multiple-year analysis of trends, and age composition of the underlying samples may partially explain differences in study findings.

Prior NYC Fitnessgram data have consistently shown decreasing, albeit varied, trends in the prevalence of obesity and severe obesity by sex, race/ethnicity, and poverty from 2006–07 to 2010–11 and 2011–12 to 2016–17 [10–12]. Conversely, the reversal of direction in obesity and severe obesity trends among Black and Hispanic students and in severe obesity among students living in impoverished areas in the current study may signify hastening disparities. For example, from 2011–12 to 2019–20, obesity prevalence increased significantly among those already experiencing the greatest burden (22.4% to 23.0% among Black students and 26.5% to 27.0% among Hispanic students). Additionally, despite a nearly universal increase among students in NYC from 2016–17 to 2019–20, the growth in prevalence occurred most rapidly among Black and Hispanic students and students living in poverty. These findings demonstrate the need for intensified efforts to combat growing inequities. Additionally, continued BMI assessment is necessary to inform how changes in activity and access to preventive care during the COVID-19 pandemic have affected disparities in childhood obesity.

Consistent with our prior analyses, data collection for this study occurred over a period during which the NYC DOHMH and DOE have continued to engage in a proactive obesity prevention and reduction strategy. This approach has included nutrition policy and education (e.g., calorie labeling, standards for city agency-provided food, media campaigns to reduce sugar-sweetened beverage consumption), healthy food availability initiatives (e.g., mobile Green Cart licenses, financial incentives for grocery stores), and guidance on active design in infrastructure [33–38]. While supporting healthy eating through adherence to state nutritional guidelines, NYC public schools also offer fitness programs, including Cooperative, Healthy, Active, Motivated and Positive Students and Move-to-Improve to promote engagement in physical activity throughout the day beyond physical education classes [39–42]. Despite limited local evidence on recent trends in childhood obesity prevalence, school districts that similarly engaged in multidisciplinary prevention approaches have previously reported declines in obesity prevalence [43]. Further, in 2019, the NYC DOHMH launched a campaign providing clinicians with resources to address pediatric obesity with families, including through referrals to food-assistance programs and behavioral interventions and promoting parent-supported healthy lifestyle choices [44]. Relatedly, the American Academy of Pediatrics recently released new evidence-based guidelines for clinically evaluating and safely and effectively treating obesity, recommending family-based interventions for children and medication and intensive lifestyle interventions for adolescents with obesity [45]. Future studies should consider changes in childhood obesity prevalence in the context of implementing these guidelines.

### Strengths and limitations

The data presented in this study provide the most recent estimates of obesity and severe obesity prevalence among NYC public school youth aged 5 to 15. As previously described, NYC Fitnessgram data have been collected for over a decade utilizing standardized training and protocols, optimizing fidelity across measurements [10, 16, 46]. Our analyses included over 5 million observations of nearly 1.4 million children, the largest number reported. The inclusion of data for school years 2011–12 through 2019–20 provides a broader time frame than previously presented, offering a more complete understanding of local changes over the past decade while providing an estimate of childhood obesity prevalence just before the COVID-19 pandemic [10–12].

Interpretation of these results should be within the context of study limitations. First, although increases in obesity and severe obesity prevalence were observed from 2016–17 to 2019–20, the repeated cross-section study design limits the ability to assess trends to the population-level rather than changes in individuals’ BMI status over time. While assessment of population-level changes in prevalence is necessary for public health surveillance, planned future analyses include the evaluation of student-level growth trajectories and changes in BMI, including transitions between BMI categories, and sociodemographic shifts in the student population over time. Relatedly, despite overall declines in obesity from 2011–12 to 2019–20, the data presented are observational and preclude establishing causal relationships with any specific intervention. Third, in 2013–14, some instructors were trained to remove an inch from the heights of students wearing shoes. Yet, for measurements flagged as being collected while the student was wearing shoes, an additional inch was subtracted from the student’s height which may have raised BMI estimates, resulting in misclassification bias. Fourth, there were many students in the 2019–20 student population for whom data were not collected due to COVID-19-related school closures, reducing coverage from >93% in each prior school year to about 56% in 2019–20 and requiring larger weights. However, the raking process used was consistent with procedures for all previous years, allowing a more reliable estimation of prevalence in the population of interest. Thus, while missingness increased from about 6% across all school years from 2011–12 to 2016–17 to over 9% from 2011–12 to 2019–20, all valid observations were weighted to be representative of the student enrollment population [10]. Still, there is limited generalizability to all NYC youth due to excluding private and charter school and special education students, representing 19.4%, 13%, and 2% of school-aged children, respectively [47]. Relatedly, changes in enrollment may partially explain increasing trends in obesity and severe obesity. For example, the proportion of students enrolled in charter schools increased from 4.4% to 11.4% over the study period. Nevertheless, the increasing trends observed may reflect true changes, suggesting that increases in BMI among NYC public school students are occurring at a higher rate than in previous years.

## Conclusions

In this study, we present the most recent estimates of obesity and severe obesity prevalence among K-8 youth attending public schools in NYC, the largest US school district, representative of over 600,000 students. We estimate that 20.9% and 6.4% of this population had obesity and severe obesity, respectively, in 2019–20. Whereas overall continued declines were observed from 2011–12 through 2019–20, consistent with prior reporting, patterns diverged across sociodemographic subgroups. Notably, while previous analyses, including data from 2011–12 through 2016–17, found larger decreases in prevalence among White and US-born students, this study showed increasing trends among Black, Hispanic, and foreign-born students. Additionally, analyses limited to 2016–17 through 2019–20 demonstrated the disproportionate burden of overall increases these students bear compared to those experienced by other groups. The growth of racial/ethnic and socioeconomic disparities and recent increasing trends signal closer alignment to trends in national data, despite persistent overall declines in obesity and severe obesity prevalence among school-aged NYC youth over the study period. These findings highlight the need for greater implementation of equity-centered obesity prevention efforts. Future research must consider the influence of the COVID-19 pandemic and changes in clinical guidance on childhood obesity and severe obesity in NYC.

## Supporting information

S1 Fig Relative risk of obesity for Hispanic, non-Hispanic Black, and Asian/Pacific Islander vs. non-Hispanic White students, grades K-8, 2006–07 to 2019–20. (TIF)

S2 Fig Relative risk of severe obesity for Hispanic, non-Hispanic Black, and Asian/Pacific Islander vs. non-Hispanic White students, grades K-8, 2006–07 to 2019–20. (TIF)
