# Supplementary figures and images for: Increasing disparities in obesity and severe obesity prevalence among public elementary and middle school students in New York City, school years 2011–12 through 2019–20
Source: PLoS One. 2024 May 15;19(5):e0302099. doi: 10.1371/journal.pone.0302099 (PMC11095699; doi:10.1371/journal.pone.0302099)

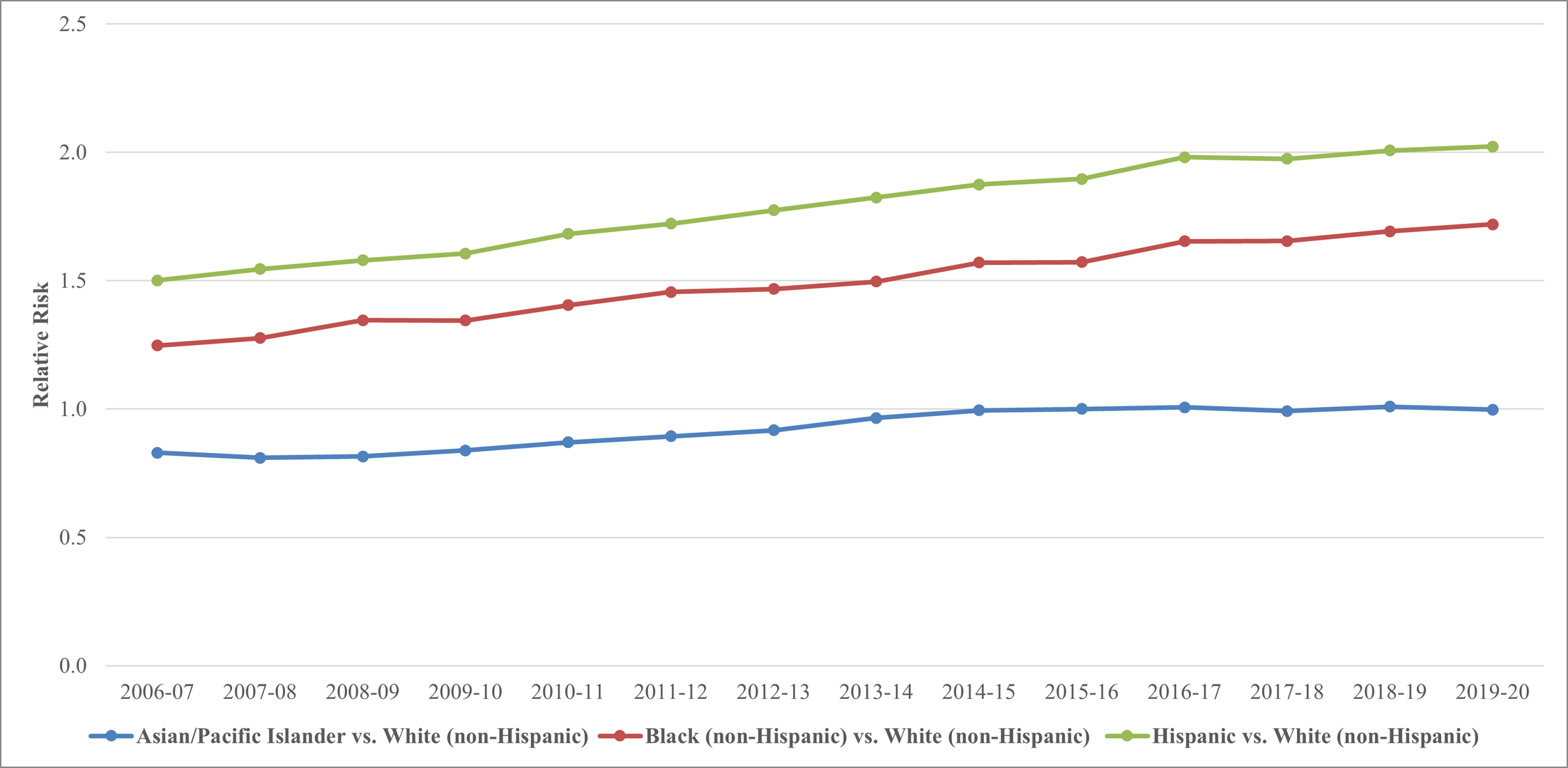

Supplement: S1 Fig — (TIF) [file pone.0302099.s001.tif]

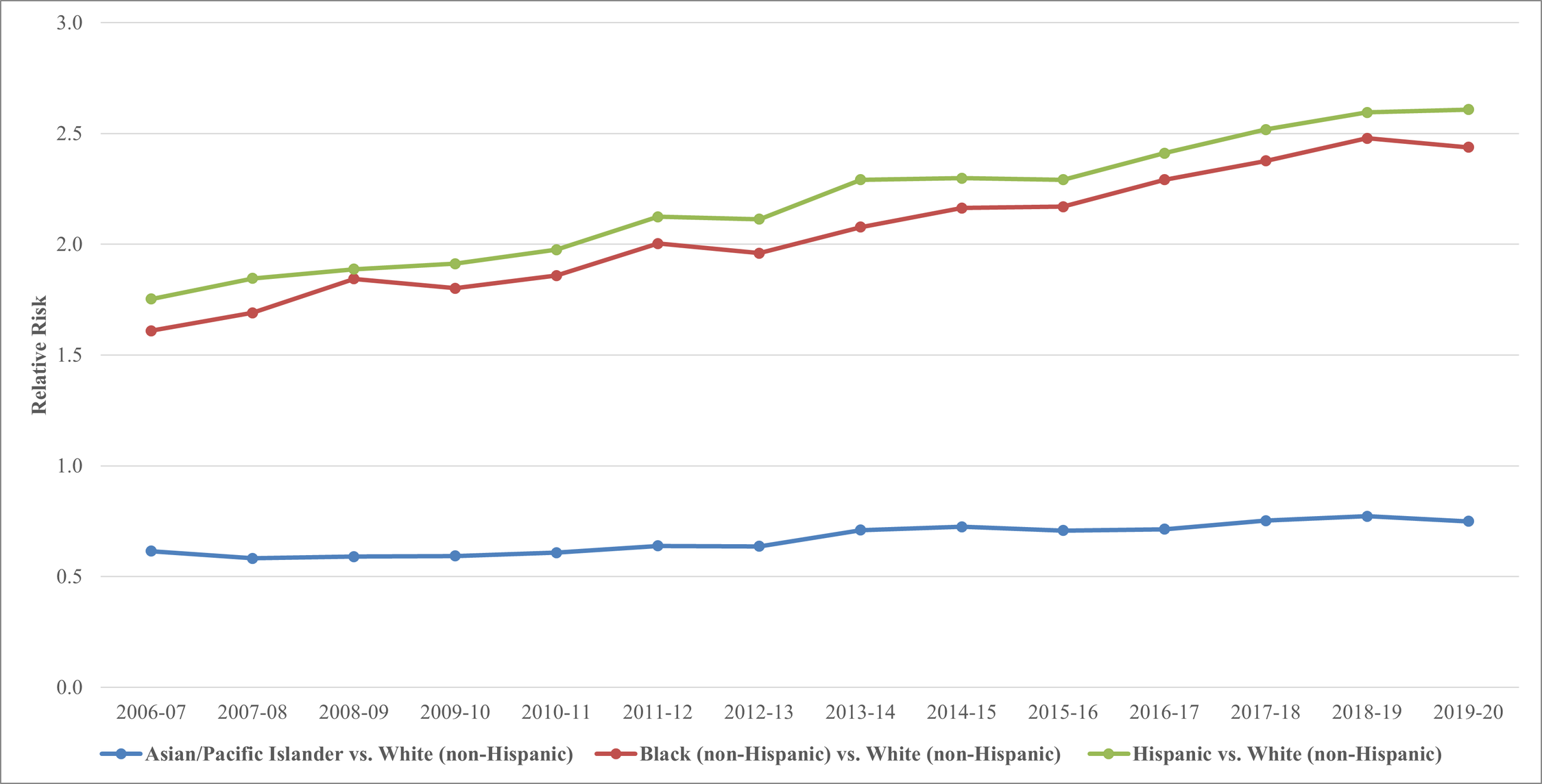

Supplement: S2 Fig — (TIF) [file pone.0302099.s002.tif]
